# Circulating *de novo* Donor Specific Antibodies and Carotid Intima-media Thickness in Pediatric Kidney Transplant Recipients, A Pilot Study

**DOI:** 10.3389/fped.2020.00017

**Published:** 2020-01-31

**Authors:** Kristen Sgambat, Sarah Clauss, Asha Moudgil

**Affiliations:** ^1^Department of Nephrology, Children's National Hospital, Washington, DC, United States; ^2^Department of Cardiology, Children's National Hospital, Washington, DC, United States

**Keywords:** donor specific antibodies, carotid intima-media thickness, cardiovascular, arteriosclerosis, pediatric, kidney transplant

## Abstract

**Background:** The presence of circulating *de novo* donor specific anti-HLA antibodies (dnDSA) has been implicated in an immune-mediated form of accelerated systemic arteriosclerosis in adult heart and kidney transplant recipients, however this has not been previously investigated in pediatric kidney transplant recipients. Carotid intima-media thickness (CIMT) is a reliable method for detection of arteriosclerosis. We hypothesized that children who develop dnDSA after kidney transplant would have increased CIMT compared with those who remain dnDSA negative.

**Methods:** A prospective, controlled pilot cohort study of 38 transplant patients and 20 healthy controls was conducted to investigate the association between CIMT and development of dnDSA after kidney transplant. CIMT, anthropometrics, blood pressure and lipid panel were measured at 1, 18, and 30 months post-transplant. DSA was checked at 6, 12, 18, 24 and 30 months post-transplant. CIMT of DSA positive transplant recipients was compared to DSA negative and controls.

**Results:** Of the 38 transplant recipients, 7 patients developed dnDSA by 18–30 months post-transplant. Among 5 dnDSA positive patients who did not receive treatment for DSA prior to CIMT measurement (n=6 observations), the median CIMT was 0.505 mm (95% CI 0.454–0.560 mm) at 18–30 months post-transplant, compared to 0.455 mm (95% CI 0.440–0.470) in DSA negative transplant recipients (*n* = 54 observations of 30 patients) and 0.450 mm (95% CI 0.436–0.460) in the healthy controls (20 observations of 20 patients). Presence of dnDSA was independently associated with a 7.8% increase in CIMT compared to those without dnDSA (p=0.006), after adjusting for race, hypertension, dyslipidemia, and abdominal obesity.

**Conclusions:** Development of dnDSA was associated with increased CIMT, an indicator of arteriosclerosis, in a cohort of dnDSA positive pediatric kidney transplant recipients. The association between dnDSA and CIMT was independent of traditional CV risk factors, including hypertension, dyslipidemia, and abdominal obesity.

## Introduction

Pediatric kidney transplant recipients are at high risk for cardiovascular (CV) disease, the second leading cause of mortality in this population (1). Traditionally, factors such as hypertension, dyslipidemia, and obesity have been associated with increased CV risk in both the general population (2) and in kidney transplant recipients (3, 4). However, recently, the presence of circulating *de novo* donor specific anti-HLA antibodies (dnDSA) have been implicated in an immune-mediated form of accelerated systemic arteriosclerosis in adult heart and kidney transplant recipients (5, 6). In addition, one pediatric study has identified a link between dnDSA and antibody-associated arteriosclerosis in heart transplant recipients (7). This form of antibody-associated arteriosclerosis is a condition characterized by arterial inflammation and fibrointimal thickening of the arteries caused by endothelial activation, a pro-inflammatory and pro-coagulant state of the endothelial cells lining the lumen of the blood vessels.

The study by Loupy et al. demonstrated that the presence of circulating dnDSA was significantly associated with severe allograft arteriosclerosis, independent of traditional CV risk factors in adult kidney transplant recipients (5). In addition, patients with dnDSA-associated arteriosclerosis had decreased allograft survival, increased occurrence of major adverse cardiovascular events, and increased mortality.

Carotid-intima media thickness (CIMT) is one known reliable indicator of arteriosclerosis that can be utilized to assess CV risk in renal transplant recipients (1, 4). The association of dnDSA with arteriosclerosis and CV risk has not been previously investigated in pediatric kidney transplant recipients. Therefore, the aim of our pilot study was to investigate the effect of dnDSA on CIMT as a marker of arteriosclerosis in pediatric kidney transplant recipients, based on the hypothesis that the presence of circulating DSA promotes vascular proliferation resulting in increased CIMT.

## Patients and Methods

### Patients and Controls

A prospective, controlled cohort study was conducted to investigate the association between CIMT and the development of dnDSA after kidney transplant. Children, 3–20 years of age, who received a kidney transplant between September 2010-January 2015 at Children's National Hospital in Washington DC were eligible to enroll in the study at the time of transplant. Patients with an estimated glomerular filtration rate (eGFR) <60 mL/min/1.73 m^2^ at 1 month post-transplant, multi-organ transplant, or nephrotic-range proteinuria were excluded from the study.

Healthy children 3–20 years of age served as controls. The healthy control participants were recruited from the local pediatric community, reflecting similar environmental exposures to the transplant cohort, and the racial distribution of the control population was matched to that of the transplant group.

Approval was obtained from the IRB at Children's National Hospital (IRB protocol number 2024). Informed consent was obtained from all participants, and the study was conducted in accordance with the Helsinki Declaration of 1975.

### Measurement and Classification of dnDSA

Transplant patients were monitored for development of dnDSA at 6, 12, 18, 24, and 30 months post-transplant, as well as for-cause. Donor-specific antibodies were tested using multiple solid phase assay platforms. In cases where flow panel reactive antibody (PRA) Screen was positive, both SAB (One Lambda, Inc., Canoga Park, CA, USA) and phenotype bead (LABScreen PRA assay by One Lamba, Inc.) assays were then performed. DSA was determined to be positive when mean fluorescence intensity (MFI) was >1000. DSA were further classified as weak if MFI was 1000–2000, strong if MFI was >2000–10,000, or very strong if MFI was >10,000.

For purposes of the study, transplant patients were classified as DSA positive (DSA+) if strong or very strong DSA was detected at 18 and/or 30 months post-transplant. Observations of CIMT at the time of DSA+ detection, prior to receiving any pharmacologic treatment for DSA or acute cellular-mediated rejection were included in the analysis. Any CIMT measurements performed after administration of treatment for DSA+ and/or acute cellular-mediated rejection, such as pulse methylprednisolone, alemtuzumab, rituximab, and bortezomib, were excluded from the analysis. CIMT measurements of patients with weak DSA (MFI >1000–2000) were also excluded from the analysis. Patients were classified as DSA negative (DSA-) if they never developed any dnDSA during the first 30 months post-transplant. In patients who were DSA+ at 18 months post-transplant but became DSA- by 30 months post-transplant, the 30 month data point was excluded from the analysis to minimize confounding effects of prior DSA positivity.

### CIMT

CIMT was measured at baseline (0–1 months post-transplant), 18, and 30 months post-transplant in the transplant group, and at a single study visit in the healthy control group. CIMT was visualized using B-mode ultrasound imaging of the arterial far wall segments of the right and left common carotid arteries and carotid bulbs. Imaging was performed according to a standard protocol by a single pediatric sonographer using aiE33 xMatrix DS Ultrasound System (Philips North America Corporation, Andover, MA, USA). Intima-media thickness of the distal, mid-, and proximal segments of right and left common carotid arteries and carotid bulbs were analyzed by a pediatric cardiologist blinded to the clinical information. A composite of the eight measured segments was then created to represent the CIMT.

### Traditional CV Risk Factors

Parameters traditionally used to evaluate CV risk, including BMI, waist-to-height ratio (WHr), fasting lipid panel, and blood pressure, were measured at 1, 18, and 30 months post-transplant in the transplant recipients. The children in the healthy control group had BMI, WHr, and blood pressure measured once, at the time of CIMT measurement. Presence of general obesity and abdominal obesity were assessed using BMI and WHr, respectively. Weight in kilograms and height in centimeters were measured and used to calculate BMI [weight (kg)/height(m^2^)]. Participants were then classified as BMI-obese if they had a BMI ≥ 95th percentile-for-sex-and-age on the Center for Disease Control (CDC) growth charts (8). Waist circumference (WC) was measured using a Gulick II fiberglass tape measure (Country Technology, Gays Mills, WI) at the upper-most lateral border of the right ileum, according to standard National Health and Nutrition Examination Study (NHANES) procedures (9). WHr was calculated as a ratio of waist in centimeters divided by height in centimeters. Participants were then classified as WHr-obese if WHr ≥ 0.5 (10).

Cut-points for abnormal lipid parameters for high density lipoprotein (HDL), low density lipoprotein (LDL), and triglycerides were defined according to 2011 Guidelines for Cardiovascular Health and Risk Reduction in Children and Adolescents for children age 3–19 (11), and by the Adult Treatment Panel III criteria for those >19 years of age (12). Dyslipidemia was defined as the presence of at least one abnormal lipid parameter (HDL, LDL, or triglyceride). Hypertension was defined by diagnosis of hypertension (systolic and/or diastolic blood pressure >95th percentile for age-sex-and -height on 3 different occasions) and requiring antihypertensive medication (13). Estimated glomerular filtration rate (eGFR) was calculated using the updated Schwartz equation for children under 18 years of age and the MDRD equation was used for those ≥ 18 years of age.

### Induction and Maintenance Immunosuppression

The transplant recipients received Thymoglobulin for induction immunosuppression, followed by maintenance immunosuppression with either with a steroid-withdrawal or steroid-based protocol, depending on their immunological risk. The steroid-withdrawal protocol was used for “standard immunological risk” patients, defined as those receiving their first transplant, with cPRA <20%, and ESRD diagnosis not due to autoimmune disorder or FSGS. The steroid-based protocol was used for “high immunological risk” patients, defined as those with history of a prior transplant, diagnosis of FSGS or autoimmune disorder, highly sensitized patients, or those with delayed graft function (as defined by need for dialysis in the immediate post-transplant period). The details of the induction therapy protocols have been previously published (14). Maintenance immunosuppression for those on the steroid-based protocol consisted of tacrolimus, mycophenolate mofetil (MMF), and prednisone, while those on the steroid-withdrawal received tacrolimus and MMF.

### Statistical Analysis

Statistical analyses were conducted using Stata 14.0 (StataCorp LP, College Station, TX, USA). Analyses of the demographic and clinical characteristics of the study and control populations were conducted using Fisher's exact test for categorical variables and Wilcoxon rank-sum for continuous variables.

**Figure 1:**
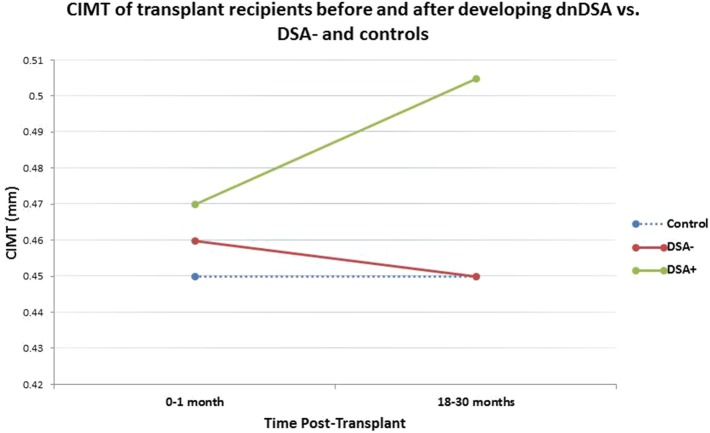
Median CIMT observations of the group who remained DSA negative decreased from 0.460 to 0.455 mm between baseline (0–1 month) and 18–30 months, while ClMT increased from 0.470 to 0.505 mm in the group who developed dnDSA at 18–30 months post-transplant. Median CIMT of healthy controls was 0.450 mm (one measurement).

The median (95% CI) of the CIMT observations of the DSA+ and DSA- transplant groups at 18–30 months post-transplant and of the control group (single time point) are presented as descriptive statistics in the Results section and in graphical form in Figure 1. In order to establish a temporal reference point, the median CIMT of the DSA+ and DSA- transplant groups are also depicted at baseline (0–1 months post-transplant). Due to the longitudinal nature of the data, bivariate statistical tests of significance were not appropriate, and therefore Figure 1 represents a descriptive presentation of the data only.

Unadjusted and adjusted linear regression models using generalized estimating equations (GEE) to account for repeated measures were used to evaluate association between CIMT and DSA status at 18–30 months post-transplant. Observations at 18 and/or 30 months post-transplant time points were included in the GEE regression analysis if both a CIMT measurement and a DSA measurement were performed at that time point. In total, 60 observations of 38 patients were included in this analysis. GEE regression analysis was selected due to its ability to account for the within-patient correlation inherent to longitudinal serial data, as well as the fact that this statistical method is robust to missing data (15). In addition, multivariable linear regression was performed at baseline (0–1 months post-transplant) to determine whether there was a pre-existing difference in CIMT between the two transplant groups prior to the onset of DSA. Due to the non-normal distribution of CIMT in the transplant group, all continuous CIMT data were log-transformed, and therefore results of the regression models are presented as a % change in CIMT. Quasi-likelihood under the independence model criterion (QIC) was used to determine the best-fit statistical model. The most robust multivariable model is reported and was adjusted for race, hypertension, dyslipidemia, and abdominal obesity by WHr.

#### Statistical Power Analysis

*Ad-hoc* power analysis indicates that the sample size of 60 observations of 38 patients provided 87% power to detect a significant association (*p* < 0.05) between dnDSA and CIMT using a 5-parameter regression model. The power analysis was based on a full model *R*^2^ = 0.52, reduced model *R*^2^ = 0.44, and change in *R*^2^ = 0.08. Therefore, the study was adequately powered to detect the primary outcome measure, a significant association between CIMT and DSA, by GEE multiple regression analysis.

## Results

### Study Population

Of 70 patients who received a kidney transplant at Children's National between September 2010 and January 2015, 61 patients met the study inclusion criteria. Of these 61 eligible patients, 43 consented to enroll in the study, and 38 of these enrolled patients completed the required study procedures. In total, 38 transplant recipients contributed 60 observations of CIMT between 18 and 30 months post-transplant to the analysis.

Within the transplant group, 18.4% of patients (7/38) had developed strong or very strong dnDSA by 18–30 months post-transplant and were thus classified as DSA+ for purposes of the study. Of the 7 DSA+ patients, 2 patients received pharmacologic treatments for DSA+ acute cellular-mediated rejection prior to their 18 month CIMT imaging, and therefore were excluded from the analysis. One additional patient had weak dnDSA detected at 18 months and was therefore excluded from the analysis. Six observations (4 observations at 18 months and 2 at 30 months post-transplant) of the remaining 5 DSA+ patients who did not receive treatments prior to CIMT measurement were included in the analysis. Of a possible 60 CIMT observations of the 30 DSA- transplant patients at 18 and 30 months post-transplant, 54 observations were included in the analysis. The 6 observations of DSA- participants were missing for the following reasons: 2 patients moved out of the area after 18 months, 2 patients missed the 18 month study visit, and 2 patients missed the 30 month study visit.

In addition, 20 healthy children contributed 20 observations to the study (one CIMT observation per each control participant).

### Characteristics of Patients and Controls

The demographics and clinical characteristics of the DSA+, DSA- and healthy control groups are summarized in Table 1. Among the transplant patients included in the dnDSA positive study group, antibody profiles included 3 patients with strong Class II, 1 with very strong Class II, and 1 with a combination of very strong Class I and II. There were no differences in the age or race distribution of the DSA+, DSA-, or control groups (*p* > 0.05). Male sex was more prevalent in the DSA- vs. the control group (*p* = 0.02), but did not differ between the DSA + and DSA – groups and also did not differ between the DSA + and control groups (*p* > 0.05). In addition, male gender was not significantly associated with CIMT in the multivariate analysis, as shown in Table 2. There were no differences in the proportion of patients with pre-emptive transplant or the mean duration of dialysis prior to transplant between DSA + and DSA- groups (*p* > 0.05). There were no statistical differences in prevalence of hypertension, dyslipidemia, BMI-obesity, abdominal obesity, steroid protocol or in eGFR between DSA+ and DSA- groups at 18 months post-transplant (*p* > 0.05).

**Table 1:** Demographics and clinical characteristics.

	**Transplant group**		**Control group**
	**DSA+**	**DSA-**	
Age at transplant (mean ± SEM)	12.5 ± 2.9 years	11.8 ± 0.8 years	11.6 ± 0.9 years
African American Race (%)	60%	53.3%	50%
Male (%)	40%	66.6%^*^	30%^*^
Duration of dialysis prior to transplant (mean ± SEM)	13.7 ± 5.6 months	23.8 ± 0.4 months	NA
Pre-emptive transplant	40%	30%	NA
Hypertension (%)^**^	60%	66.6%	0%
Dyslipidemia (%)^**^	20%	30%	NA
BMI-Obesity (%) ^**^	50%	36.7%	0%
Abdominal obesity (%)^**^ (WHr≥0.5)	60.0%	53.3%	0%
Steroid protocol (%)‡	60.0%	53.3%	NA
eGFR at 18 months (mean ± SEM)	96.4± 14.8 ml/min/1.73m^2^	96.6 ± 5.1 ml/min/1.73 m^2^	NA
dnDSA	➢60% strong Class II ➢ 20% very strong Class II ➢ 20% very strong Class I and II	No DSA	NA

Categorical variables were compared by Fisher's exact test and continuous variables by Wilcoxon rank-sum. There were no differences in the age or race distribution of the DSA+, DSA-, or control groups (p > 0.05). Male sex was more prevalent in the DSA- vs. the control group (p = 0.02

* *), but did not differ between the DSA + and DSA – groups and also did not differ between the DSA + and control groups (p > 0.05). There were no differences in any of the remaining variables between DSA + and DSA- groups (p > 0.05)*.

** *Proportion of patients affected at any point during the 18–30 month post-transplant time period*. *‡Indicates proportion of patients who received maintenance steroid therapy*.

**Table 2:** Association of variables with CIMT (unadjusted analysis).

**Variable**	**% change in CIMT^a^ (95% CI)**	***p*-value**
dnDSA	10.2% (3.3 to 17.1%)	0.03^*^
African American race	9.4% (6.2 to 12.6%)	0.0001^*^
Hypertension	1.4% (−2.0 to 4.5%)	0.42
Dyslipidemia	−1.1% (−3.7 to 1.4%)	0.17
Obesity by BMI	−3.9% (−7.1 to 1.0%)	0.13
Abdominal Obesity (WHr)	0.23% (−3.1 to 3.5%)	0.89
Dialysis duration	0.08% (−0.03 to 0.20%)	0.16
eGFR	−0.02% (−0.07 to 0.03%)	0.43
Steroid therapy	−1.4% (−5.6 to 2.7%)	0.51
Male sex	−1.0% (−5.7 to 3.6%)	0.66
Age	−0.4% (−3.7 to 2.9%)	0.80

* *Significant association with CIMT by GEE linear regression, without adjusting for any covariates*.

a *Due to non-normal distribution, continuous CIMT data were log-transformed, and therefore results are presented as a % change in CIMT*.

### CIMT

At 18–30 months post-transplant, DSA+ transplant recipients had median CIMT of 0.505 mm (95% CI 0.454–0.560 mm), compared to 0.455 mm (95% CI 0.440–0.470) in DSA- transplant recipients and 0.450 mm (95% CI 0.436–0.460) in the healthy controls. In order to visualize a temporal relationship between DSA and CIMT, we also examined the CIMT of the DSA+ and DSA- groups at baseline (0–1 months post-transplant), prior to development of DSA. As shown in Figure 1, the median CIMT of the group who remained DSA- during the first 30 months post-transplant decreased slightly from 0.460 mm (95% CI 0.430–0.490) to 0.455 mm (95% CI 0.440–0.470) between baseline and 18–30 months post-transplant, while the median of CIMT of the group who became DSA+ increased from 0.47 mm (95% CI 0.440–0.491) at baseline to 0.505 mm (95% CI 0.454–0.560 mm) over the same time period. Due to the longitudinal nature of the data, bivariate statistical comparison was not appropriate. As these CIMT comparisons are descriptive only in nature and do not account for other potentially confounding factors, unadjusted and adjusted multivariable regression analyses were performed.

## Association of DSA With CIMT

### 18–30 Months Post-transplant, Unadjusted Analysis

Results of the unadjusted GEE regression analysis showed that the presence of DSA and African American race were associated with increased CIMT in transplant recipients. As shown in Table 2, DSA positive transplant recipients had 10.2% thicker CIMT compared with DSA negative transplant recipients (*p* = 0.03), and CIMT was 9.4% thicker in the African American transplant recipients compared with non-African Americans (*p* = 0.0001), prior to adjusting for any covariates. The remaining variables, which included BMI-obesity, WHr-obesity, hypertension, dyslipidemia, sex, age, steroid therapy, and eGFR at the time of CIMT measurement were not significantly associated with CIMT in the unadjusted analysis. Duration of dialysis prior to transplant was also not significantly associated with CIMT at 18–30 months post-transplant.

### 18–30 Months Post-transplant, Adjusted Analysis

After adjusting for potential confounding factors, multivariable GEE analysis showed that the presence of DSA was independently associated with a 7.8% increase in CIMT compared to those transplant recipients without DSA (*p* = 0.006). In addition, African American race was independently associated with a 9.3% increase in CIMT among transplant recipients (*p* = 0.0001). As presented in Table 3, the best-fit multivariable model was adjusted for race, abdominal obesity, hypertension, and dyslipidemia. Abdominal obesity was associated with a 3.7% increase in CIMT, with borderline statistical significance (*p* = 0.059).

**Table 3:** Association post-transplant dnDSA with CIMT (adjusted analysis).

**Variable**	**% change in CIMT^a^ (95% CI)**	***p*-value**
dnDSA	7.8% (2.2 to 13.4%)	0.006^*^
African American race	9.3% (5.5 to 13.1%)	0.0001^*^
Abdominal Obesity (WHr)	3.7% (0.0 to 7.5%)	0.059
Hypertension	0.70% (−2.8 to 4.2%)	0.69
Dyslipidemia	−1.6% (−5.2 to 2.0%)	0.38

* *Significant association with CIMT by GEE linear regression, adjusted for race, hypertension, dyslipidemia, and abdominal obesity by WHr (p <0.05)*.

a *Due to non-normal distribution, continuous CIMT data were log-transformed, and therefore results are presented as a % change in CIMT*.

### Temporal Validation of Relationship of DSA and CIMT, Adjusted Analysis

Multivariable linear regression was also performed to establish a timeline of the relationship between DSA status and CIMT among the transplant recipients. Results of this analysis showed that at baseline, prior to development of any DSA, neither group of transplant recipients had increased CIMT (coefficient −0.7%, 95% CI −6.3 – 4.0%, *p* = 0.81). As in the 18–30 month analysis, this linear regression model was adjusted for race, abdominal obesity, hypertension, and dyslipidemia. The results of this analysis demonstrate that association of increase in CIMT with DSA+ patients did in fact develop after the onset of dnDSA, and not before.

## Discussion

This pilot study is the first to demonstrate that development of dnDSA is associated with an indicator of arteriosclerosis, evidenced by increased CIMT, in a cohort of DSA positive pediatric kidney transplant recipients. Transplant patients with dnDSA exhibited thicker CIMT in comparison to transplant recipients without dnDSA at 18–30 months post-transplant, while CIMT of DSA- transplant recipients was similar to that of healthy controls. The association between dnDSA and CIMT was independent of traditional CV risk factors, including hypertension, dyslipidemia, and abdominal obesity. Our data also showed a temporal relationship between development of dnDSA and increased CIMT, as the association was not present at the time of transplant, prior to development of dnDSA.

Antibody-associated arteriosclerosis was previously described by Loupy et al. in a population of adult kidney transplant recipients (5). This study showed that presence of circulating dnDSA was associated with arterial inflammation and complement fraction C4d deposition resulting in intimal thickening of the arteries, and higher levels of DSA correlated with greater severity of arteriosclerosis (5).

Our findings are also supported by studies of dnDSA-associated vasculopathy in adult and pediatric heart transplant recipients (6, 7), which showed that circulating dnDSA were associated with increased risk of coronary artery vasculopathy, allograft failure, and mortality. The mechanism of action for accelerated antibody-associated arteriosclerosis has been previously demonstrated in animal models, as direct administration of anti-HLA antibodies to vascular endothelium induces release of inflammatory cytokines and complement activation, resulting in intimal hyperplasia and vasculopathy (16, 17).

The study by Loupy et al. also showed that adults with antibody-associated arteriosclerosis had 2.5 and 4.1 fold increased risk of major adverse CV events compared to those with non-antibody associated arteriosclerosis and those without significant arteriosclerosis, respectively, independent of other CV risk factors. The authors hypothesized that complement activation and release of inflammatory agents triggered by circulating dnDSA trigger not only injury localized to the allograft, but also ignite a generalized systemic process of arteriosclerosis, similar to that seen in other autoimmune diseases (5).

As major adverse CV events are not common in children, we used CIMT, a well-validated indicator for the early detection of CV disease, to identify arteriosclerosis in our study population. It was interesting to note that at the time of transplant, all transplant recipients had similar CIMT. In the group that went on to develop dnDSA, CIMT increased from 0.470 mm at baseline to 0.505 mm at 18–30 months post-transplant, while the DSA negative group had a slight decrease in CIMT from 0.460 to 0.455 mm (similar to the median CIMT of healthy controls, 0.450 mm) over the same time period (Figure 1). Previous longitudinal cohort studies of CIMT in children with ESRD suggest that CIMT would be expected to remain stable or slightly improve over time after kidney transplantation (18, 19). The results of our study suggest that the DSA negative transplant recipients follow the expected pattern, exhibiting stable or slightly improved CIMT, achieving a median CIMT matching that of race-matched healthy controls by 18–30 months post-transplant, while children who develop dnDSA exhibit an increasing trend in CIMT over time post-transplant.

Our results indicate that CIMT of DSA+ transplant recipients is approximately 0.05 mm higher than those of DSA- patients and healthy controls. Due to the rarity of major CV events in children, the exact magnitude of increase in CIMT that corresponds to increase in CV risk is not known. However, literature indicates that in adults, each absolute increase in cIMT by 0.1 mm corresponds to an increase in risk of stroke by 13–18% and an increase in risk of myocardial infarction by 10–15% (20). Therefore, based on this data combined with meta-analyses of pediatric studies of CIMT, an absolute increase in of 0.05 mm in CIMT a child is expected to be clinically significant in terms of portending future CV risk (21). The lack of significant association between CIMT with hypertension, dyslipidemia, and BMI that was observed in our study is in agreement with that of other pediatric solid organ transplant populations, as reported in a recent meta-analysis (21). Abdominal obesity as measured by WHr demonstrated a stronger magnitude of association with increased CIMT among our transplant cohort than did BMI, with borderline statistical significance (*p* = 0.059). Prior studies suggest that WHr is likely a more sensitive indicator of CV risk than BMI in children with kidney disease (22), but further research is needed to investigate its relationship to CIMT.

Strengths of our study included a prospective study design with a healthy control group that was matched by race to the transplant recipients. Matched race distribution between the study and control groups is important due to the known association of increased CIMT in African American children compared to other races (23). This was a pilot study, and as such was limited by small sample size, particularly in the DSA+ group. Our study was sufficiently powered to detect the association between DSA and CIMT, however larger multicenter studies would be needed to investigate more granular associations such as the type and strength of anti-HLA antibodies with severity of arteriosclerosis. In addition, although we demonstrated a temporal relationship between DSA development and increased CIMT, we cannot prove causality in the absence of histologic evidence. Our study design did incorporate several measures to isolate the possible effects of dnDSA on CIMT from those of the treatments given to patients with dnDSA. We excluded CIMT measurements from DSA+ patients who had already received therapeutic interventions for DSA with concomitant acute cell mediated rejection. In addition, our statistical analysis controlled for possible side effects of maintenance steroid therapy, including obesity, dyslipidemia and hypertension during the first 30 months post-transplant. Larger studies of longer duration post-transplant would be needed to examine possible long-term effects of dnDSA on cardiovascular risk profile in patients with persistent DSA.

In summary, dnDSA was found to be associated with increased CIMT in a cohort of pediatric kidney transplant recipients. The novel finding identified in our pilot study should be followed up by larger studies to verify this association and to elucidate the mechanism, effects, and possible therapies to treat antibody-associated arteriosclerosis in kidney transplant recipients.

## Data Availability Statement

The raw data supporting the conclusions of this article will be made available by the authors, without undue reservation, to any qualified researcher.

## Ethics Statement

The studies involving human participants were reviewed and approved by Children's National Hospital Institutional Review Board. Written informed consent to participate in this study was provided by the participants' legal guardian/next of kin.

## Author Contributions

All authors (KS, SC, AM) contributed to the conception, design, data collection and analysis, as well as writing and revision of the manuscript for this study. In addition, AM, pediatric nephrologist and Director of Kidney Transplantation, was the PI of the study and oversaw all of the research and regulatory aspects of the study. Statistical analysis was performed by KS, and analysis of CIMT was performed by pediatric cardiologist, SC.

### Conflict of Interest

The authors declare that the research was conducted in the absence of any commercial or financial relationships that could be construed as a potential conflict of interest.

## Abbreviations

CV: Cardiovascular
dnDSA: *De novo* donor specific antibody
DSA+: Donor specific antibody positive
DSA-: Donor specific antibody negative
CIMT: Carotid intima media thickness
eGFR: Estimated glomerular filtration rate
PRA: Panel reactive antibody
MFI: Mean fluorescence intensity
BMI: Body mass index
WHr: Waist to height ratio
CDC: Center for disease control
NHANES: National Health and Nutrition Examination Survey
HDL: High density lipoprotein
LDL: Low density lipoprotein
WC: Waist circumference
GEE: Generalized estimating equation
QIC: Quasi-likelihood under the independence model criterion
ESRD: End stage renal disease.
